# Nocturia as a clinical marker of loss of function and resilience or risk factor for frailty in older adults? Results of the Berlin Aging Study II

**DOI:** 10.1007/s11357-025-01525-9

**Published:** 2025-01-31

**Authors:** Maximilian König, Carolin Malsch, Joany Mariño, Valentin Max Vetter, Yulia Komleva, Ilja Demuth, Elisabeth Steinhagen-Thiessen

**Affiliations:** 1 Department of Internal Medicine and Geriatrics, University Medicine Greifswald, Walther-Rathenau-Str. 49, Greifswald, 17475 Germany; 2 Geriatric Medicine Center, Kreiskrankenhaus Wolgast, Wolgast, Germany; 3 Department of Biomathematics and Statistics, Institute of Mathematics and Computer Science, University of Greifswald, Greifswald, Germany; 4 Department of Internal Medicine B, University Medicine Greifswald, Greifswald, Germany; 5 Department of Endocrinology and Metabolic Diseases (including Lipid Metabolism), Charité-Universitätsmedizin Berlin, corporate member of Freie Universität Berlin and Humboldt-Universität zu Berlin, Biology of Aging Working Group, Berlin, Germany; 6 Medical Department of Endocrinology and Metabolic Diseaseas (including Lipid Metabolism), Charité-Universitätsmedizin Berlin, Berlin, Germany; 7 BCRT-Berlin Institute of Health Center for Regenerative Therapies, Charité-Universitätsmedizin Berlin, corporate member of Freie Universität Berlin and Humboldt-Universität zu Berlin, Berlin, Germany

**Keywords:** Frailty, Nocturia, Circadian rhythm, Aging, Longitudinal

## Abstract

The current study examined cross-sectional and longitudinal associations between nocturia and frailty in a cohort of men and women aged 60 years and older, as evidence on this topic was lacking. We analyzed baseline and follow-up data (*n* = 1671) from the Berlin Aging Study II (BASE-II), a prospective longitudinal cohort study focusing on the factors associated with “healthy” vs. “unhealthy” aging. Self-reported nocturia was dichotomized into < / ≥ 2 micturitions per night, and frailty was assessed using the Fried frailty phenotype. Covariables were identified a priori based on a review of the existing literature. At baseline, 70.2% of the participants were robust, 28.9% were pre-frail, and 0.9% were frail; 254 participants (23.6%) had self-reported nocturia. In longitudinal analyses, the prevalence and incidence of frailty at follow-up significantly increased when nocturia was present at baseline. Over a median follow-up of 7.1 years, there were 41 incident frailty cases (IR 5.6, 95%-CI 3.9–7.2 per 1000 person-years). After adjusting for age, sex, morbidity burden, and baseline frailty status, baseline nocturia was associated with a 2.23-fold increased risk (95%-CI 1.17–4.18) of frailty at follow-up. Nocturia is associated with an increased risk of developing or progressing in frailty in older adults, and may serve as an early clinical marker for the progression of frailty.

**Supplementary Information:**

The online version contains supplementary material available at 10.1007/s11357-025-01525-9.

## Introduction

With demographic change, the proportion of old and very old people continues to increase. Unfortunately, healthspan is not keeping pace with the increase in life expectancy, so age-associated diseases, geriatric syndromes, and frailty are becoming increasingly relevant [1–3]. Frailty is a clinically identifiable state of diminished physiological reserve and increased vulnerability [4]. Given its high prevalence in older adults and strong association with adverse outcomes and increased healthcare utilization, identifying and addressing frailty is of utmost importance [5]. Further understanding of the nature, trajectories, and mechanisms driving its development is paramount for designing effective interventions to reduce the frailty burden in older people [6].

 Ferrucci et al. have proposed a hierarchical model in which different metrics of aging—biological, phenotypic, and functional—progress along distinct trajectories with time lags [7]. In healthy adults, multiple physiological systems interact harmoniously as part of a complex dynamic system to maintain allostasis and homeostasis. However, biological aging—shaped by interactions with diverse factors, including psychological, lifestyle, genetic, environmental, and disease-related influences—leads to phenotypic changes, reduced system efficiency, and impaired inter-system communication. Physical frailty represents a critical threshold where these declines and dysregulations exceed resilience capacity[8].

 Identifying when biological and phenotypic resilience mechanisms are on the verge of exhaustion, signaling the transition to functional ageing and frailty, remains a challenge. One potential indicator of such impending multisystem failure, that has received limited attention is nocturia. Nocturia is when a person wakes up several times during the night to urinate [9]. It is the leading cause of sleep disturbance in older adults [10]. Fewer younger adults report nocturia (< 5%), but it affects about half of older adults aged 60 years and older and about 80% of those aged 80 years and older [11]. Nocturia has a significant impact on overall health and quality of life [10], being associated with daytime dysfunction and sleepiness, fractures, reduced gait speed, reduced functionality, and mobility, among others [8, 11]. Two or more nocturia episodes have been shown to be a marker of poor health in older women [13]. However, nocturia, like sleep in general, is often under-recognized in everyday clinical practice, research and public health agendas [14], and its relationship to frailty is not fully explored. The existing evidence on the association between nocturia and frailty is scarce and contradictory [15]. Both cross-sectional and longitudinal studies have found evidence for an association between sleep disturbances and frailty [16–19]. And indeed, there is some preliminary evidence showing that circadian disruption associated with artificial light at night and nocturia may play a role in development of frailty [20]. However, there are often design, methological, and analysis constraints, limiting the value of the existing studies in disentangling this complex relationship [21]. Studies that would allow causal inference, such as longitudinal studies that meet the necessary quality criteria, are still lacking.

We hypothesize that nocturia may serve as an early clinical marker of functional decline and reduced resilience, preceding the onset of the clinical frailty phenotype. It may reflect subclinical impairments in multiple physiological systems, such as circadian regulation, renal function, and fluid homeostasis, and their complex interplay—essentially acting as an early indicator of the broader multisystem dysregulation seen in frailty. Furthermore, beyond being a marker, nocturia likely contributes actively to the progression of frailty through its disruptive effects on sleep, metabolism, and overall homeostasis. Either way, understanding the temporal link between nocturia and frailty is of significant interest. If nocturia precedes frailty, it could provide a critical window for early intervention. Moreover, recognizing nocturia as an early marker may help identify at-risk individuals, prompting a comprehensive evaluation of other aging-related domains and vulnerabilities.

Using longitudinal data from the Berlin Aging Study II, we aimed to examine cross-sectional and longitudinal associations between nocturia, frailty, and mortality.

## Methods

### Study population and design

The Berlin Aging Study II (BASE-II) is a prospective cohort study investigating factors associated with “healthy” and “unhealthy” aging, and has been described in detail previously [22, 23]. Participants were community-dwelling, comparably well-functioning older adults (aged between 60 and 84 years), and a control group of young adults. The baseline medical assessments took place between 2009 and 2014. Follow-up data from 1083 BASE-II participants were obtained as part of the GendAge study [24] between 2018 and 2020, and were complemented by information on vital status received from the civil register. Our analysis used data from the baseline assessments of the older age group in BASE-II and the corresponding follow-up assessments in GendAge.

The study was approved by the ethics committee of the Charité-Universitätsmedizin Berlin (EA2/029/09 and EA2/144/16) and conducted according to the declaration of Helsinki. All participants gave written informed consent for study participation.

### Nocturia assessment

Nocturia was assessed using the same two questions, both at baseline and follow-up: “Do you have to get up at night to urinate?” and “How often do you need to urinate per night?” We operationalized nocturia as two or more episodes of nocturia. [25].

### Mortality

The vital status was periodically checked at the civil register, most recently in May 2023.

### Frailty assessment

Frailty was operationalized according to the phenotype concept proposed by Fried et al. [26]. As previously described, minor modifications to the original methodology were necessary to adjust for small differences in how the criteria were assessed in BASE-II [27]. The five frailty phenotype criteria were assessed as follows:

- “Unintentional weight loss”: Unintentional loss of at least 5% of body weight during the past year.
- “Self-reported exhaustion”: two questions from the “Center for Epidemiological Studies depression” scale [28].
- “Weakness”: low handgrip strength measured by Smedley Dynamometer (Scandidact, Denmark). We used the original cut-off values as suggested by Fried et al. [26].
- “Slow walking speed”: walking speed was assessed in the Timed Up & Go test [29].
- “Low physical activity”: was assessed with the question “Are you seldom or never physically active?”

According to the number of components present, participants were categorized as frail (3–5), prefrail (1–2), or robust (0) [26].

### Covariables

Sleep was assessed using the Pittsburgh Sleep Quality Index, a widely used tool in clinical practice and research for assessing subjective sleep habits and quality [30]. The questionnaire consists of 19 questions evaluating subjective sleep quality, latency to fall asleep, sleep duration, sleep efficiency, sleep disorders, use of sleep medication, and impairment of daytime activity. Regarding subjective sleep quality, we grouped the categories "poor" and "very poor" to create the variable poor subjective sleep quality. Unfortunately, the subjective sleep quality question was unavailable at follow-up.

All other diagnoses were recorded in a structured manner as part of the data collection process, integrating information from various sources. These were used to compute a morbidity index largely based on the categories of the Charlson comorbidity index, which is a weighted sum of moderate to severe, mostly chronic physical illnesses: myocardial infarction, congestive heart failure, peripheral vascular disease, cerebrovascular disease, ulcer disease, chronic pulmonary disease, connective tissue disease, mild liver disease, diabetes, diabetes with end-organ damage, renal disease, hemiplegia, lymphoma, leukemia, any tumor, and moderate to severe liver disease. The weighting was adopted from the original publication (Charlson et al., 1987) for these domains [31, 32].

Further self-reported information included in the analysis were falls and hospitalization in the past 12 months prior to the baseline and follow-up assessments, respectively.

### Statistical analyses

Participant’s characteristics were compared between strata using a t-test, one-way analysis of variance (ANOVA), Kruskal‒Wallis test, Wilcoxon rank sum test, Fisher’s test, or χ^2^ test, as appropriate.

The main outcomes of interest were frailty and mortality, while the exposure was nocturia. Hence, we calculated Kaplan–Meier curves and mortality rates per 1000 person-years (PY) with 95% percentile bootstrapping confidence intervals, stratified by nocturia and frailty status at baseline. Incidence rates of frailty per 1000 PY with percentile bootstrapping confidence intervals to a level of 95% were calculated stratified for baseline nocturia.

As the duration of follow-up was heterogeneous between participants, we analyzed the relationship between nocturia and incident frailty through (1) a Poisson regression with the duration of observation as an offset in the regression function, and (2) adjusting for the duration of observation in the logistic regression (among other confounders). Incidence rate ratios (IRRs) and odds ratios (ORs) with 95% bootstrapping confidence intervals (95% CIs) are presented after adjusting for key confounders (age, sex, frailty status at baseline, and morbidity index). Frailty transitions were visualized in an alluvial plot. A comprehensive assessment of the predictors of frailty was beyond this study’s aim; thus, we limited our models to including the variables mentioned above.

Participants, who were lost to follow-up and those with missing data for the key exposures and outcomes were not included in the longitudinal analyses with frailty as the outcome**.**

We modelled the transitions between robust, pre-frail, frail, and death with a multi-state Markov model using the R package msm [33]. This model takes into account the competing risk of death, time-varying covariates (age, nocturia and morbidity) and heterogeneous follow-up times due to its specification for intermittently observed data. We report the intensities for the transitions and the respective hazard ratios for the covariates, together with their confidence intervals.

All analyses were performed using Stata SE 18.0 (StataCorp, College Station, TX) and R (version 2024.09.0 + 375 [34]), The alluvial plot was created with the ggplot2 [35] and ggalluvial packages [36].

### Strobe statement

The manuscript was prepared in compliance with the STrengthening the Reporting of Observational Studies in Epidemiology (STROBE) statement [37].

## Results

### Study population

**Fig. 1:**
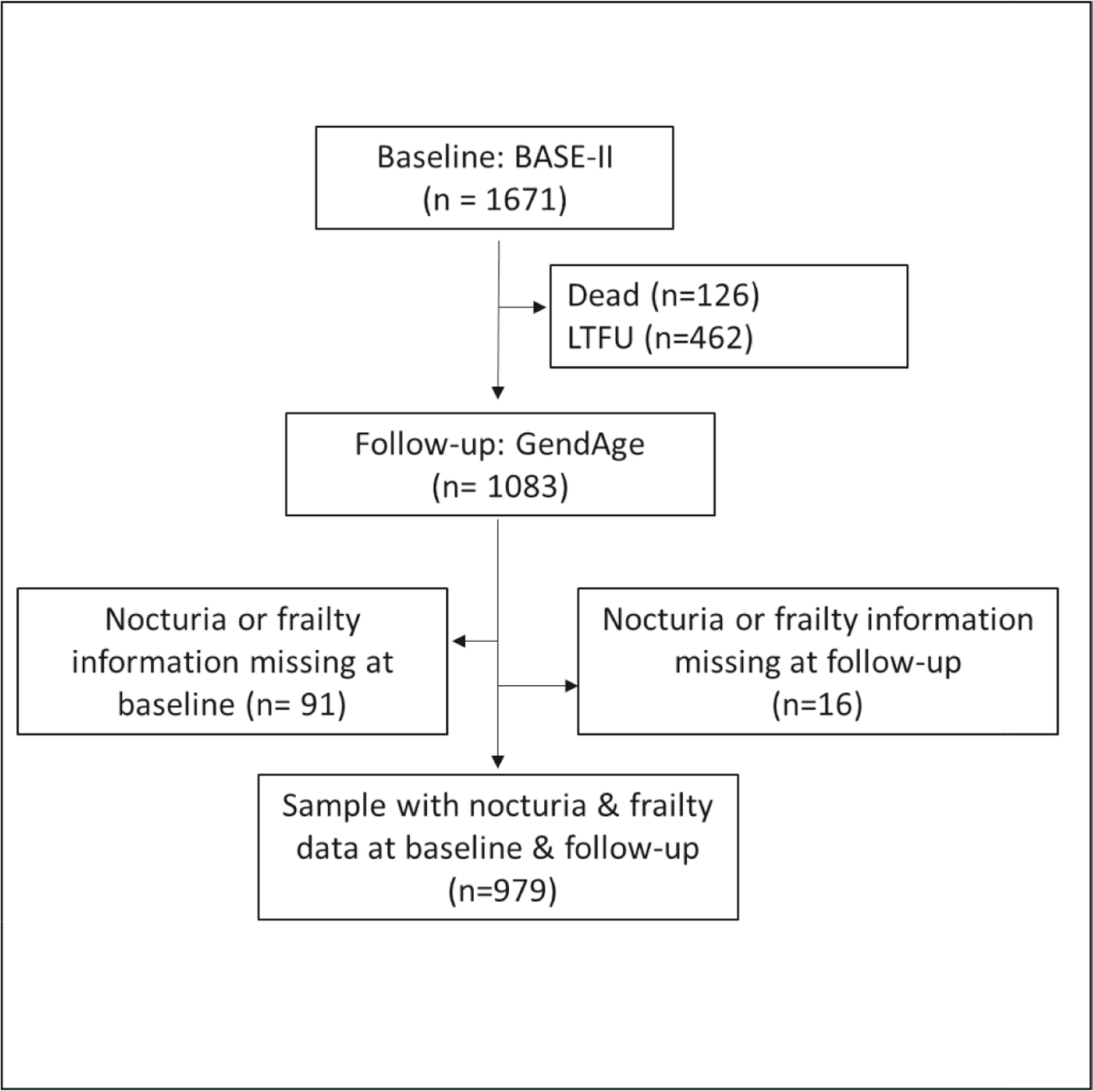
Flowchart showing the derivation of the study population. *GendAge is a subsample of BASE-II participants. BASE-II, Berlin Aging Study II, LTFU, loss to follow-up

Figure 1 illustrates the sampling procedure. The original BASE-II sample included 1671 participants aged 60 years and older (range: 60–84 years). At follow-up, 1083 participants (64.8%) of the original BASE-II sample were seen at a median of 7.1 years (IQR 6.2–8.7; range: 3.91–10.37 years) after the baseline examination (Supplementary Fig. 1). 126 participants (7.5%) died between baseline and follow-up. 462 participants (27.6%) were lost to follow-up, but were alive according to information from the civil register by March 3, 2023 (end of follow-up assessments). A further 110 participants died after follow-up, giving a total of 236 out of 1671 participants (14.1%) who had died by May 2023. The characteristics of the full baseline sample and the reduced subsample with available follow-up data are presented in Table 1. Participants not seen at the follow-up (*n* = 588, 35.2%) were older, more often frail or pre-frail, and more likely to suffer from nocturia at baseline (Supplementary Table 1).

**Table 1:** Characteristics of participants in the BASE-II study and the GendAge subsample

	Original BASE-II *n* = 1671	GendAge subsample *n* = 1083	
	Baseline	Baseline	Follow-up
Age, mean (SD)	68.3 (3.7)	67.8 (3.5)	75.6 (3.8)
Female sex, *n* (%)	862 (51.6)	563 (52.0)	-
Nocturia, *n* (%)	422 (25.4)	254 (23.6)	423 (39.2)
Frailty, *n* (%)			
Robust	1029 (68.1)	700 (70.2)	502 (46.9)
Pre-frail	469 (31.0)	288 (28.9)	520 (48.6)
Frail	14 (0.9)	9 (0.9)	48 (4.5)
Morbidity index, mean (SD)	1.01 (1.23)	0.97 (1.20)	1.39 (1.54)
Fallen in the past 12 months, *n* (%)	508 (30.6)	325 (30.1)	237 (27.3)
Hospitalizations in the past 12 months, *n* (%)	290 (17.4)	183 (16.9)	246 (23.1)
Subjective sleep duration in hours, median (IQR)	7 (6–8)	7 (6–8)	7 (6–8)
Poor sleep quality (subjective), *n* (%)	256 (16.7)	171 (17.2)	-

Data are presented as mean (standard deviation, SD), number of observations (percentage), or median (IQR = 25th,75th percentile)

### Associations between nocturia, frailty, and mortality

The mortality rate per 1000 PY was 16.9 (95%-CI 14.8–19.0). Stratification by baseline nocturia and frailty status revealed significant differences in mortality rates. Mortality was significantly higher in participants with nocturia compared to those without, and in participants with frailty or pre-frailty compared to robust participants (Table 2, Supplementary Figs. 2a-b).

**Table 2:** Mortality rates per 1000 person-years (PY), total and stratified by baseline nocturia and frailty status at baseline (*n* = 1671)

	Mortality rate per 1000 PY (95%-CI)
Total	16.9 (14.8–19.0)
Nocturia, yes	22.8 (18.2–27.6)
Nocturia, no	15.0 (12.7–17.3)
Frail	51.3 (11.5–114.0)
Pre-frail	20.9 (16.7–25. 4)
Robust	14.1 (11.8–16.4)

* PY, *Person years, *95%-CI*, 95%-confidence interval

In multivariable analysis, frailty at baseline remained significantly associated with death (Table 3). Further independent associations were found for age and sex, but not for pre-frailty, nocturia, and overall morbidity burden.

**Table 3:** Multivariable-adjusted^#^ Rate Ratios and Odds Ratios of death in BASE-II (*n* = 1671)

	Rate ratio (95%-CI)	Odds ratio (95%-CI)
Pre-frail*	1.42 (0.74–2.66)	1.44 (0.73–2.77)
Frail*	**8.80 (1.40–30.30)**	**8.89 (1.20–43.90)**
Nocturia**	0.69 (0.30–1.42)	0.68 (0.29–1.44)
Baseline age	**1.13 (1.04–1.22)**	**1.14 (1.05–1.24)**
Female sex	**0.41 (0.21–0.76)**	**0.44 (0.22–0.86)**
Morbidity index	0.89 (0.67–1.14)	0.87 (0.64–1.13)
Follow-up duration	-	0.94 (0.75–1.18)

* reference: robust, **reference: no nocturia at baseline, ^#^adjusted for baseline age, sex, morbidity index and follow-up duration

### Associations between nocturia and frailty

**Fig. 2:**
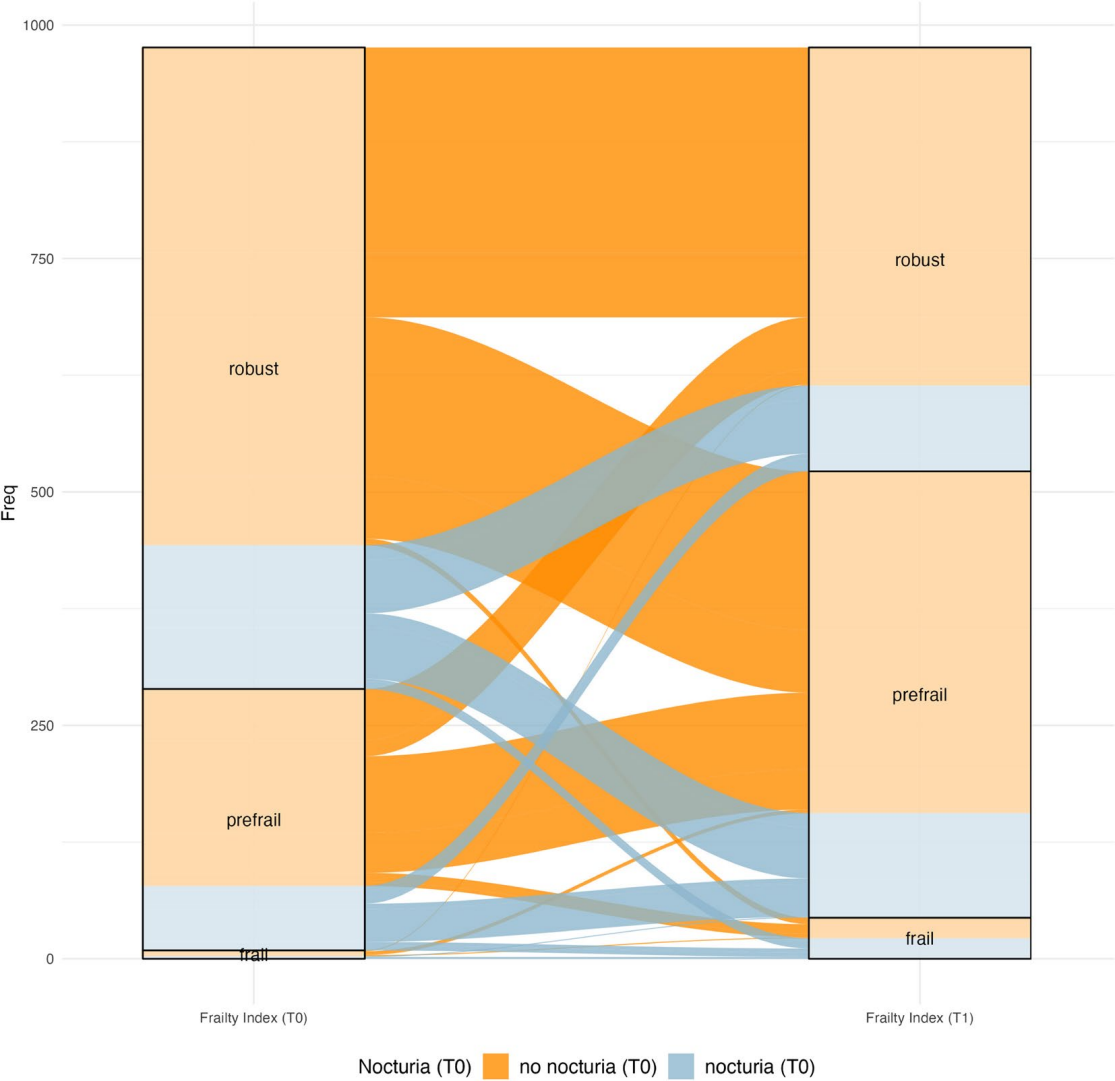
Alluvial plot showing frailty transitions (robust ↔ pre-frail ↔ frail) stratified by nocturia status at baseline (*n* = 979). At baseline, 689 (70.4%) were robust, 281 (28.7%) pre-frail, and 9 (0.9%) frail. By follow-up, 456 (46.6%) were robust, 479 (48.9%) were pre-frail, and 44 (4.5%) were frail. Of those initially robust, 308 (44.7%) transitioned to pre-frailty, 18 (2.6%) to frailty, while 363 (52.7%) stayed robust. Nocturia was associated with higher probabilities of transitioning to pre-frailty (45.2% vs. 44.6%) and frailty (7.1% vs. 1.3%), and a lower probability of remaining robust (47.7% vs. 54.1%) by follow-up. Among the 281 initially pre-frail participants, 92 (32.7%) returned to robust, 166 (59.1%) remained pre-frail, and 23 (8.2%) progressed to frailty. Nocturia reduced the probability of becoming robust (27.5% vs. 34.4%), while increasing the probability of transitioning to frailty (13.0% vs. 6.6%). Of the 9 participants who were initially frail, 1 (11.1%) returned to robust, 5 (55.6%) became pre-frail, and 3 (33.3%) remained frail at follow-up. Of the 44 frail participants at follow-up, 18 (40.9%) had been robust and 23 (52.3%) pre-frail at baseline. Notably, among 41 new frailty cases, nocturia was present in 11 (61.1%) of those initially robust and 9 (39.1%) of those initially pre-frail

Nocturia prevalence increased from 23.6% at baseline to 39.2% at follow-up (Table 1). Prevalence of pre-frailty and frailty increased from 28.9% and 0.9% to 48.6% and 4.5%, respectively. At follow-up, 350 (35.6%) participants had progressed in their frailty status, with 41 (4.2%) new cases of frailty. Figure 2 illustrates the transitions in frailty status from baseline to follow-up stratified by nocturia status. Nocturia was associated with higher probabilities of transitioning from robust to pre-frailty (47.7% vs. 44.6%) and frailty (7.1% vs. 1.3%), and a lower probability of remaining robust (47.7% vs. 54.1%) by follow-up. Likewise, nocturia reduced the likelihood of returning to robust (27.5% vs. 34.4%), while increasing the risk of transitioning from pre-frailty to frailty (13.0% vs. 6.6%).

A cross-sectional analysis revealed no significant association between nocturia and frailty at baseline (*p* = 0.633), whereas a significant association was identified at follow-up, where frailty and pre-frailty were significantly more prevalent in participants with nocturia than in those without (5.8% vs. 3.7%, and 53.3% vs. 45.7%, respectively; *p* = 0.005).

Longitudinally, participants with nocturia at baseline showed a significantly higher frailty incidence rate (11.8, 95%-CI 7.1–17.0 per 1000 PY) than participants without nocturia at baseline (3.8, 95%-CI 2.3–5.4 per 1000 PY). According to multivariable Poisson regression adjusted for baseline age, sex, morbidity index, and baseline frailty status, nocturia at baseline was associated with a 2.23-fold increased frailty rate at follow-up (95%-CI 1.17–4.18, Table 4). This result was consistent with multivariable logistic regression, which showed 2.45-fold (95%-CI 1.23–4.83) increased odds of frailty at follow-up when nocturia was present at baseline. Similar results were observed for frailty incidence, with a 2.07-fold increased frailty incidence rate per 1000 PY (95%-CI 1.05–4.00) and 2.23-fold increased odds of incident frailty (95%-CI 1.10–4.43) for participants with nocturia at baseline compared to those without nocturia.

**Table 4:** Association between nocturia at baseline and follow-up frailty (*n* = 915)

	Rate ratio (95%-CI)	Odds ratio (95%-CI)
Frailty at follow-up		
Nocturia	2.23 (1.17–4.18)	2.45 (1.23–4.83)
Age at baseline	1.11 (1.02–1.20)	1.12 (1.02–1.22)
Female sex	1.21 (0.65–2.31)	1.14 (0.57–2.32)
Morbidity index at baseline	1.42 (1.16–1.72)	1.53 (1.22–1.90)
Frailty at baseline	3.26 (1.86–5.65)	3.89 (2.12–7.26)
Follow-up duration	-	1.33 (1.04–1.70)
Incident frailty at follow-up		
Nocturia	2.07 (1.05–4.00)	2.23 (1.10–4.43)
Age at baseline	1.13 (1.03–1.23)	1.14 (1.04–1.26)
Female sex	1.32 (0.69–2.58)	1.27 (0.63–2.61)
Morbidity index at baseline	1.53 (1.24–1.86)	1.62 (1.29–2.03)
Follow-up duration	-	1.29 (1.01–1.66)

95%-*CI*, 95% confidence interval

In addition, we used multi-state modelling to analyze the effect of nocturia and other covariates on the transitions between the states “robust”, “pre-frail”, “frail”, and “death” taking into account the censored frailty transition time points as well as the competing risk of death and time-varying covariates (age, nocturia and morbidity). The observed transition frequencies and estimated transition intensities are presented in Table 5.

**Table 5:** Observed transition frequencies (percentage relative to the full sample), and *estimated transition intensities (95%-CI)* from the multi-state model

To state From state	Robust	Pre-frail	Frail	Death
Robust	365 (30.5%) *−0.16 (−0.20, −0.13)*	309 (25.8%) *0.14 (0.12, 0.18)*	18 (1.5%) 0*	105 (8.8%) *0.02 (0.01, 0.03)*
Pre-frail	93 (7.8%) *0.10 (0.06, 0.13)*	167 (13.9%) *−0.14 (−0.22, −0.09)*	23 (1.9%) *0.03 (0.01, 0.17)*	101 (8.4%) *0.02 (0.01, 0.02)*
Frail	1 (0.1%) 0*	5 (0.4%) *0.28 (0.04, 1.95)*	3 (0.3%) *−0.29 (−1.9, −0.04)*	8 (0.7%) *0.01 (0.00, 1.43)*

*The model assumes that transitions to frailty must pass through the pre-frailty stage, whether or not this stage is explicitly observed. As a result, direct transitions between robust and frail states were not estimated, even though they were occasionally observed in the data

**Fig. 3:**
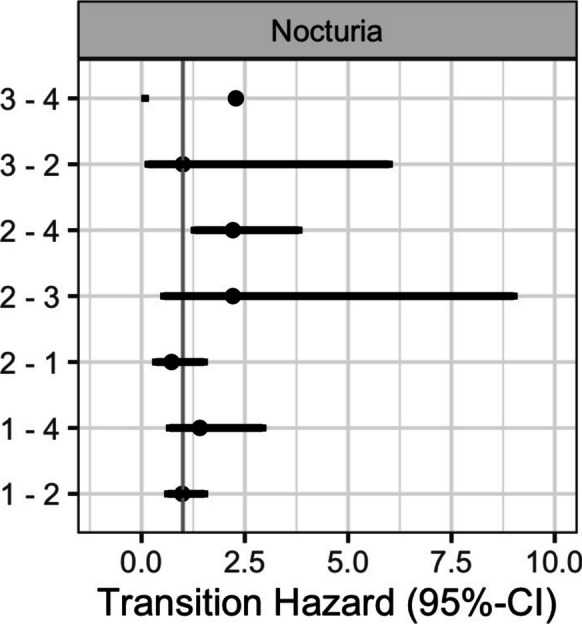
Effect of nocturia on state transitions in the multi-state model (HR and 95%-CI). 1 = robust, 2 = pre-frail, 3 = frail, 4 = death. Note: The upper level of the confidence interval for the transition from frail to death could not be displayed due to the wide confidence interval (0.09–60.72)

The estimated transition hazard ratios for all covariates are shown in Supplementary Table 4. Figure 3 highlights the effect of nocturia on state transitions in the multi-state model, while a more comprehensive illustration of the multi-state model is provided in Supplementary Fig. 3. Nocturia increased the hazard of transitioning from pre-frail to death by a factor of 2.21 (95% CI: 1.28–3.80). Higher morbidity increased the hazard of transitioning from pre-frail to frail by 2.18 (95% CI: 1.29–3.69). Additionally, age influenced several transitions, including from robust to death, pre-frail to death, and pre-frail to frail.

To complete the emerging clinical picture, the clinical characteristics of participants at both time points and stratified by nocturia and frailty status are presented in Supplementary Tables 2 and 3. Participants with nocturia were consistently older, had higher hospitalization rates, and exhibited more overall morbidity than those without nocturia at both time points. No significant sex difference was observed at baseline; however, at follow-up, nocturia was more prevalent among male participants. Self-reported sleep duration was shorter in people with nocturia, and more people with nocturia reported poor sleep quality at baseline. With increasing frailty, participants reported poor sleep quality more often at both time points.

## Discussion

The aim of this study was to examine both cross-sectional and longitudinal associations between nocturia, frailty, and mortality, given the limited and heterogeneous evidence from prior cross-sectional studies. We found that individuals with nocturia at baseline had significantly higher prevalence and incidence of frailty at follow-up. These findings were consistent across absolute numbers, frailty incidence rates, and multivariable adjusted models, including a multi-state model adjusted for age, sex, and morbidity burden. To the best of our knowledge, our findings in this longitudinal study are the first to confirm that nocturia is associated with an increased risk of frailty and frailty progression. Nocturia did not emerge as a significant independent predictor of mortality in multivariable regression but increased the mortality hazard for pre-frail individuals in the multi-state model. Our findings further reinforce the established association between frailty and mortality, as demonstrated through multivariate regression models and a multi-state model.

### Limitations

Before interpreting our results further, we identify and discuss the potential sources of bias, imprecision, or confounding that could have influenced our findings.

Mortality: the most reliable analysis in this study pertains to the risk factors for death, as it includes all participants in BASE-II regardless of their participation in the follow-up. We obtained the needed vital status information from the civil register, thus mitigating the impact of an incomplete follow-up.

Selection bias: from baseline (*n* = 1671) to follow-up (*n* = 1083), 126 (7.5%) participants died and 462 (27.6%) were lost to follow-up. It is likely that participants lost to follow-up were not missing at random, as they tended to be older, more often frail or pre-frail, and tended to have a higher morbidity burden at baseline (Supplementary Table 1). Consequently, our analyses may underestimate the true association between nocturia and frailty.

Competing risk of death: participants who died after the baseline assessment could not be included in the follow-up. However, death as competing risk factor could not be accounted for in the Poisson analysis. Since the BASE-II design relied on two cross-sectional surveys without time-to-event data for frailty, Cox regression accounting for competing risk of death was not possible. Consequently, our regression analyses may yield biased estimates of the association between nocturia and frailty, as they do not consider the impact of mortality on the development of frailty.

Frailty transitions: frailty was assessed at baseline and again at follow-up; however, there may be shorter-term, or even repeated transitions in frailty status. For a more accurate approach, it would be desirable to measure frailty at several fixed intervals after baseline, which would give a clearer picture of the frailty development in the population.

Variable follow-up times: the heterogeneous follow-up times (Supplementary Fig. 1) pose a challenge in interpreting the relationship between nocturia and frailty. If the relationship is indeed causal, the varying durations of nocturia exposure could influence the observed effect. This variability in exposure time might lead to inconsistencies in the effect of nocturia on frailty between participants. Nevertheless, the observation period, with a median of 7.1 years (IQR 6.2–8.7), is a valuable strength of this study, allowing potential causal factors sufficient time to at least partially manifest their effects.

We used a multi-state model to analyze the effect of nocturia on the transitions between robust, pre-frailty, frailty and death, while accounting for the heterogeneous follow-up times, competing risk of death and time-varying covariates. The model assumes that transitions to frailty must pass through the pre-frailty stage, whether or not this stage is explicitly observed. As a result, direct transitions between robust and frail states were not estimated, even though they were occasionally observed in the data. As shown in Table 5, the frequencies of these transitions (*) were relatively low, suggesting that their omission is unlikely to significantly impact the validity of our findings in addressing the research question. While this assumption aligns with the model's design and the conceptual framework of frailty progression, it represents a simplification that should be considered when interpreting the results. Overall, the performance of the multi-state model was moderate, as reflected in some notably wide confidence intervals. A larger sample size, particularly with an increased number of frail patients, would be necessary to achieve a more robust and satisfactory model.

Self-reported data: many variables in this study, including nocturia, sleep duration, and sleep quality, were self-reported. These self-reported data introduce the possibility of recall and information bias, potentially leading to misclassification and, consequently, to under- or overestimation of the strength of the association between the exposures and outcomes.

Sample size: BASE-II was designed to identify and characterize factors associated with ‘healthy’ vs. ‘unhealthy’ ageing [22] and participants were of above-average health (as reflected in the low morbidity burden) and education [22, 23]. Consequently, our sample has a low frailty prevalence, limiting the ability to model this subgroup comprehensively. Aside from that, we observed reasonable confidence interval widths in most of our analyses.

Generalizability: it has been shown that nocturia is more prevalent in ethnic minorities and people with lower socioeconomic status [38], while BASE-II participants were mostly of European ancestry and educated above average. This selection bias limits the generalizability of our findings to populations other than the source population. The comparatively low prevalence of frailty and nocturia at both time points may also be explained by the above-average health (reflected in the low morbidity burden) and education of BASE-II participants [23].

### Interpretation

#### Nocturia, frailty, and associated comorbidities in older adults

As anticipated, the prevalence of pre-frailty, frailty, and nocturia increased over the more than seven-year follow-up period in our study. However, compared to other cohorts, the prevalence and incidence of frailty and nocturia were relatively low, both at baseline and follow-up [11, 25, 39]. Previous studies have suggested that in older age groups (60 years old and older), nocturia becomes more frequent in men compared to women [9]. However, we found that nocturia was about equally distributed in men and women at baseline, which may imply that sex-specific etiologies underlying nocturia, such as benign prostatic hyperplasia or pelvic floor problems, are unlikely to play a major role at this time, whereas at follow-up, nocturia was slightly but significantly more prevalent in men than in women.

Participants with nocturia were consistently older, had higher hospitalization frequencies, and exhibited higher overall morbidity than those without nocturia at both time points. Both frailty and nocturia at baseline were associated with more sleep disturbances and lower subjective sleep duration and quality. These observations are consistent with previous research [25, 40, 41]. These factors may not only contribute to the development or progression of frailty but can be responsible for progressive decline and adverse outcome [26].

#### Nocturia as an early marker of circadian disruption and frailty in aging

Nocturia is closely intertwined with sleep disruption and disturbed circadian rhythm, which may be a central mechanistic link between nocturia and frailty in older adults [15]. Circadian rhythm is central to homeostasis, and its disruption affects several physiological processes and signaling pathways, resulting, for example, in metabolic dysfunction and increased inflammation, which are hallmarks of the frailty phenotype [42–45]. While frailty has been described as a transition from a harmonious balance to “cacophonous” chaos, the links between circadian rhythms, their disruption, and frailty remain largely unexplored [8, 46]. Based on our findings, which show a temporal sequence of nocturia and frailty, we suggest that nocturia may indicate a transition to functional ageing and impending frailty, due to the exhaustion of biological and phenotypic resilience mechanisms, and multisystem dysregulation. In this sense, nocturia may serve as a functional biomarker of physiological decline and impaired circadian rhythm, and as a predictor of adverse frailty trajectories. In addition, nocturia can even contribute to a worsening of dysregulation by maintaining and intensifying sleep–wake disturbances.

Indeed, aging per se is associated with a weakening in the amplitude of the circadian rhythm, and many lifestyle factors and living conditions in old age have the potential to exacerbate a decline of the circadian rhythm, such as loss of daily structure due to retirement, less time outdoors due to difficulty walking, and less locomotor capacity and activity, among others [15, 47].

In this context, the shift-worker example is instructive: shift workers are regularly affected by both nocturia and premature frailty [15]: first the circadian rhythm is disturbed, then nocturia occurs and finally frailty—the temporal sequence is very clear, even if the time latency is considerable. This leads to the conclusion that circadian optimization may offer an actionable, simple and holistic therapeutic approach [48]. Appropriate circadian-based strategies, rhythm-stabilizing or reinforcing interventions, be they timed exercise, hormonal therapies (e.g. desmopressin for nocturic polyuria) or behavioral strategies (e.g. timing of fluid intake, timed meals, light exposure), have the potential to both reduce nocturia and delay or prevent the transition to frailty, and may also benefit the ageing process via physiology, metabolism and behavior beyond nocturia and frailty [15].

### Outlook

Nocturia and frailty are highly relevant clinical problems that require increased attention and research – not least because affected people experience high levels of suffering [9].

Our findings need to be confirmed and substantiated in future longitudinal studies, ideally with prospective data collection. Larger sample sizes, including a sufficient number of frail individuals, are necessary to obtain unbiased and precise estimates using advanced statistical methods. The competing risk of death should be considered early in the study design, along with more frequent measurements of both nocturia and frailty to better capture transitions. Future research could also explore the use of alternative biological aging markers, such as epigenetic clocks.

It will be valuable to investigate whether nocturia is a true risk factor or simply an indicator of broader dysregulation. Future work in both humans and translational research, including animal and preclinical models of frailty, should investigate the relationship between circadian rhythmicity, nocturia, and frailty [49]. These studies should utilize interventions that may either mitigate or exacerbate circadian disruption, nocturia and frailty [15]. If causality is established, future research should explore whether consistent treatment of nocturia (e.g. via circadian optimization) can delay, prevent or reverse existing frailty or pre-frailty in adequately powered, randomized, controlled studies.

## Conclusions

This study adds to the growing body of evidence linking nocturia to the onset and progression of frailty, and suggests its potential role as an early clinical marker of functional decline and reduced resilience. Although the present study already provides valuable insights, future research with more targeted designs and fixed follow-up intervals is needed to deepen our understanding of the proposed associations, especially if the aim is to investigate causality. A better understanding will ultimately contribute to a more targeted approach to improving the quality of life of older adults.

## Supplementary Information

Below is the link to the electronic supplementary material. 

ESM1 (DOCX 248 KB)

## Data Availability

Due to concerns for participant privacy, data are available only upon reasonable request. Please contact the scientific coordinator Ludmila Müller at lmueller@mpib-berlin.mpg.de for additional information.
