# Supplementary material for: Nocturia as a clinical marker of loss of function and resilience or risk factor for frailty in older adults? Results of the Berlin Aging Study II
Source: GeroScience. 2025 Jan 31;47(3):4139–51. doi: 10.1007/s11357-025-01525-9 (PMC12181548; doi:10.1007/s11357-025-01525-9)
Supplement: Supplementary file 1 — (DOCX 248 KB) [file 11357_2025_1525_MOESM1_ESM.docx]

**Supplementary material**

## Nocturia as a clinical marker of loss of function and resilience or risk factor for frailty in older adults? Results of the Berlin Aging Study II

Maximilian König^1,2,#^, Carolin Malsch^3,#^, Joany Mariño^4^, Valentin Max Vetter^5^, Yulia Komleva^6^, Ilja Demuth^5,8,*^, and Elisabeth Steinhagen-Thiessen^7*^

^1^Department of Internal Medicine and Geriatrics, University Medicine Greifswald, Germany

^2^Geriatric Medicine Center, Kreiskrankenhaus Wolgast, Wolgast, Germany

^3^Department of Biomathematics and Statistics, Institute of Mathematics and Computer Science, University of Greifswald, Greifswald, Germany

^4^Department of Internal Medicine B, University Medicine Greifswald, Germany

^5^Department of Endocrinology and Metabolic Diseases (including Division of Lipid Metabolism), Biology of Aging working group, Charité-Universitätsmedizin Berlin, corporate member of Freie Universität Berlin and Humboldt-Universität zu Berlin, Berlin, Germany

^6^Department of Internal Medicine and Geriatrics, University Medicine Greifswald, Germany

^7^Medical Department of Endocrinology, Diabetes and Metabolic Medicine, Charité-Universitätsmedizin Berlin, Berlin, Germany

^8^BCRT-Berlin Institute of Health Center for Regenerative Therapies, Charité-Universitätsmedizin Berlin, corporate member of Freie Universität Berlin and Humboldt-Universität zu Berlin, Berlin, Germany

^#^,* Contributed equally

**Supplementary Table 1.** Characteristics of participants included and excluded from the analysis.

|  | **Included**  1083 (64.8%) | **Excluded***  588 (35.2%) | **p-value** |
| --- | --- | --- | --- |
| Age, mean (SD) | 67.8 (3.5) | 69.2 (3.9) | <0.001 |
| Female sex, n (%) | 563 (52.0) | 299 (50.9) | 0.695 |
| Nocturia, n (%) | 254 (23.6) | 168 (28.7) | 0.027 |
| Frailty at baseline,  n (%) |  |  | 0.043 |
| Robust | 700 (70.2) | 329 (63.9) |  |
| Pre-frail | 288 (28.9) | 181 (35.1) |  |
| Frail | 9 (0.9) | 5 (1.0) |  |
| Morbidity index at baseline,  median (IQR) | 0.97 (1.20) | 1.09 (1.28) | 0.069 |
| Fallen in the past 12 months, n (%) | 325 (30.1) | 183 (31.4) | 0.615 |
| Hospitalizations in the past 12 months, n (%) | 183 (16.9) | 107 (18.4) | 0.502 |
| Subjective sleep duration in hours, median (IQR) | 7 (6-8) | 7 (6-8) | 0.782 |
| Poor sleep quality (subj.), n (%) | 171 (17.2) | 85 (15.7) | 0.497 |

*Notes and abbreviations:* Data are presented as mean (standard deviation, SD), number of observations (percentage), or median (IQR = 25th,75th percentile); *excluded due to death or loss to follow-up.

**Supplementary Table 2.** Participants’ characteristics at baseline and follow-up, stratified by nocturia status (n=1083).

| **Baseline*** | | | | | | | | | |  |
| --- | --- | --- | --- | --- | --- | --- | --- | --- | --- | --- |
|  | | | **No Nocturia**  821 (75.8%) | **Nocturia***  254 (23.5%) | **Missings**  8 (0.7%) | | | **p-value** | |  |
| Age, years | | | 67.6 (3.5) | 68.5 (3.3) | 0 | | | <0.001 | |  |
| Female sex, % | | | 438 (53.3) | 122 (48.0) | 0 | | | 0.158 | |  |
| Frailty at T0, % | | |  |  | 86 | | | 0.633 | |  |
| - Robust | | | 539 (70.9) | 158 (68.4) |  | | |  | |  |
| - Pre-frail | | | 215 (28.3) | 70 (30.3) |  | | |  | |  |
| - Frail | | | 6 (0.8) | 3 (1.3) |  | | |  | |  |
| Fallen in the past 12 months, % | | | 250 (30.6) | 74 (29.1) | 4 | | | 0.723 | |  |
| Hospitalizations in the past 12 months, % | | | 122 (14.9) | 61 (24.0) | 1 | | | 0.001 | |  |
| Morbidity index | | | 0.91 (1.16) | 1.15 (1.32) | 97 | | | <0.001 | |  |
| Subjective sleep duration, hours | | | 7 (6.5-8) | 7 (6-8) | 91 | | | 0.015 | |  |
| Poor sleep quality (subj.), % | | | 104 (13.8) | 67 (28.8) | 91 | | | <0.001 | |  |
| **Follow-up**** | | | | | | | | | |  |
|  | **No Nocturia**  656 (60.6%) | | | **Nocturia***  423 (39.1%) | | **Missings**  4 (0.4%) | **p-value** | | | |
| Age, years | | 75.3 (3.7) | | 76.1 (3.8) | | 0 | <0.001 | | | |
| Female sex, % | | | 362 (55.2) | 200 (47.3) | 0 | | | | 0.013 |  |
| Frailty T1, % | | |  |  | 13 | | | | 0.005 |  |
| - Robust | | | 330 (50.6) | 170 (41.0) |  | | | |  |  |
| - Pre-frail | | | 298 (45.7) | 221 (53.3) |  | | | |  |  |
| - Frail | | | 24 (3.7) | 24 (5.8) |  | | | |  |  |
| Fallen in the past 12 months, % | | | 131 (25.1) | 106 (30.7) | 215 | | | | 0.084 |  |
| Hospitalizations in the past 12 months, % | | | 127 (19.8) | 117 (28.0) | 19 | | | | 0.002 |  |
| Morbidity index | | 1.32 (1.53) | | 1.52 (1.55) | | 143 | 0.058 | | | |

*Notes and abbreviations:* *Nocturia at baseline defined as ≥2 episodes/night; ** nocturia at follow-up; Data are presented as mean (standard deviation), number of observations (percentage), or median (IQR = 25^th^,75^th^ percentile).

**Supplementary Table 3**. Participants’ characteristics at baseline and follow-up, stratified by frailty status (n=1083).

| **Baseline*** | | | | | |  |
| --- | --- | --- | --- | --- | --- | --- |
|  | **Robust**  700 (64.6%) | **Pre-frail**  288 (26.6%) | **Frail**  9 (0.8%) | **Missings**  86 (7.9%) | **p-value** |  |
| Age, years | 67.60 (3.29) | 68.21 (3.92) | 67.00 (3.97) | 0 | 0.146 | |
| Female sex, % | 355 (50.7) | 155 (53.8) | 5 (55.6) | 0 | 0.656 |  |
| Nocturia, % | 158 (22.7) | 70 (24.6) | 3 (33.3) | 8 | 0.663 |  |
| Fallen in the past 12 months, % | 211 (30.2) | 90 (31.2) | 2 (22.2) | 4 | 0.823 |  |
| Hospitalization in the past 12 months, % | 120 (17.1) | 45 (15.6) | 2 (22.2) | 1 | 0.766 |  |
| Morbidity index | 0.88 (1.10) | 1.16 (1.33) | 1.67 (1.41) | 97 | 0.005 |  |
| Subjective sleep duration, hours | 7 (6.5-8) | 7 (6-8) | 8.3 (7.3-8.9) | 91 | 0.003 |  |
| Poor sleep quality (subj.), % | 93 (14.3) | 64 (24.3) | 1 (16.7) | 91 | 0.001 |  |
| **Follow-up**** | | | | | |  |
|  | **Robust**  502 (46.4%) | **Pre-frail**  520 (48.0%) | **Frail**  48 (4.4%) | **Missings**  13 (1.2%) | **p-value** |  |
| Age, years | 75.09 (3.76) | 75.96 (3.60) | 77.45 (4.55) | 0 | <0.001 |  |
| Female sex, % | 254 (50.6) | 276 (53.1) | 28 (58.3) | 0 | 0.497 |  |
| Nocturia, % | 170 (34.0) | 221 (42.6) | 24 (50.0) | 4 | 0.005 |  |
| Fallen in the past 12 months, % | 95 (24.4) | 127 (29.4) | 12 (32.4) | 215 | 0.207 |  |
| Hospitalization in the past 12 months, % | 104 (21.1) | 124 (24.3) | 15 (31.2) | 19 | 0.187 |  |
| Morbidity index | 1.22 (1.41) | 1.45 (1.54) | 2.62 (2.16) | 143 | <0.001 |  |

*Notes and abbreviations:* *Frailty at baseline; ** Frailty at follow-up; Data are presented as mean (standard deviation), numbers(proportions), or median(IQR = 25^th^,75^th^ percentile).

**Supplementary Table 4.** Transition hazards with 95% confidence intervals for nocturia, age, sex, and morbidity.

| Transition | Nocturia | Age | Sex | Morbidity |
| --- | --- | --- | --- | --- |
| Robust - prefrail | 0.99 (0.64-1.52) | 1.04 (0.96-1.12) | 0.77 (0.51-1.16) | 1.09 (0.91-1.30) |
| Robust - death | 1.41 (0.68-2.93) | **1.17 (1.03-1.34)** | 0.87 (0.44-1.70) | 1.14 (0.88-1.48) |
| Prefrail - robust | 0.73 (0.35-1.51) | 0.98 (0.88-1.09) | 0.66 (0.36-1.24) | 0.95 (0.72-1.25) |
| Prefrail - frail | 2.21 (0.54-9.01) | **1.13 (1.00-1.27)** | 0.19 (0.01-4.90) | **2.18 (1.29-3.69)** |
| Prefrail - death | **2.21 (1.28-3.80)** | **1.36 (1.25-1.48)** | 0.62 (0.35-1.07) | 1.16 (0.97-1.40) |
| Frail - prefrail | 1.00 (0.17-5.99) | 1.06 (0.96-1.17) | 0.07 (0.00-2.23) | 1.60 (0.87-2.93) |
| Frail - death | 2.28 (0.09-60.72) | 0.63 (0.33-1.20) | 0.26 (0.02-3.44) | 1.21 (0.61-2.41) |

**Supplementary figure 1**.


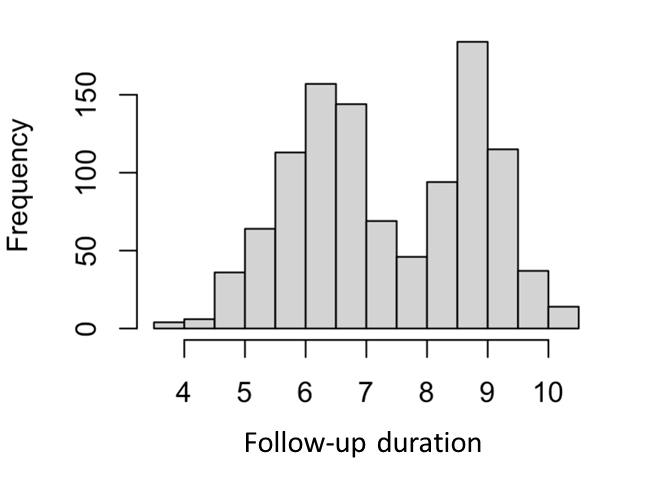


**Supplementary figure 1:** Bi-modal distribution of observed follow-up duration (in years).

**Supplementary figure 2a.**

**
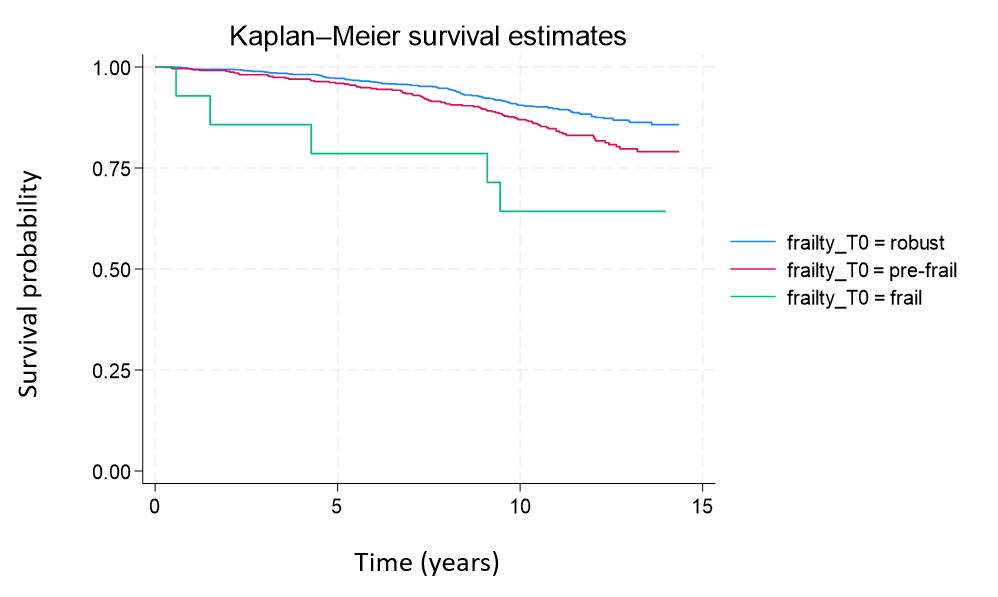
**

**Supplementary figure 2b.**


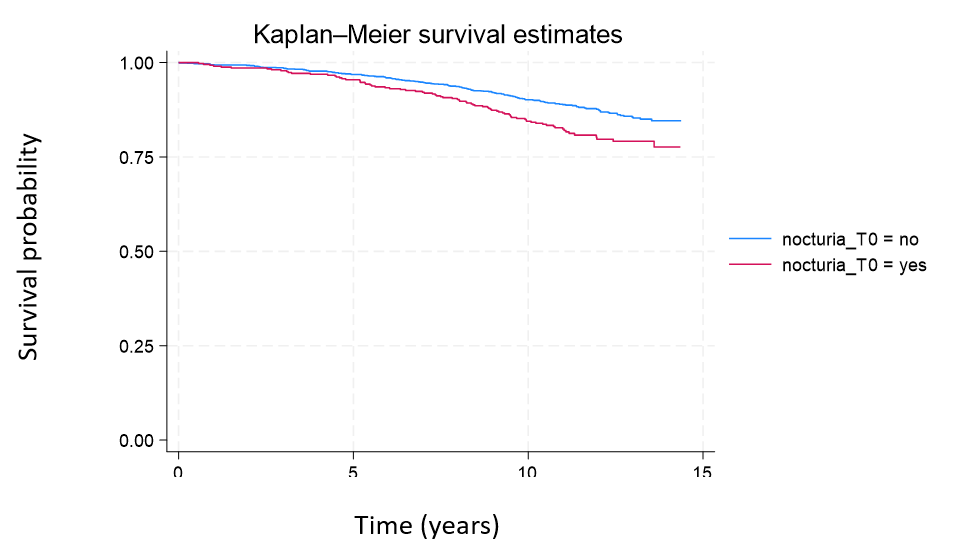


**Supplementary figures 2a-b:** Kaplan-Meier survival estimates for BASE-II (n=1671) by a) frailty status and b) nocturia.


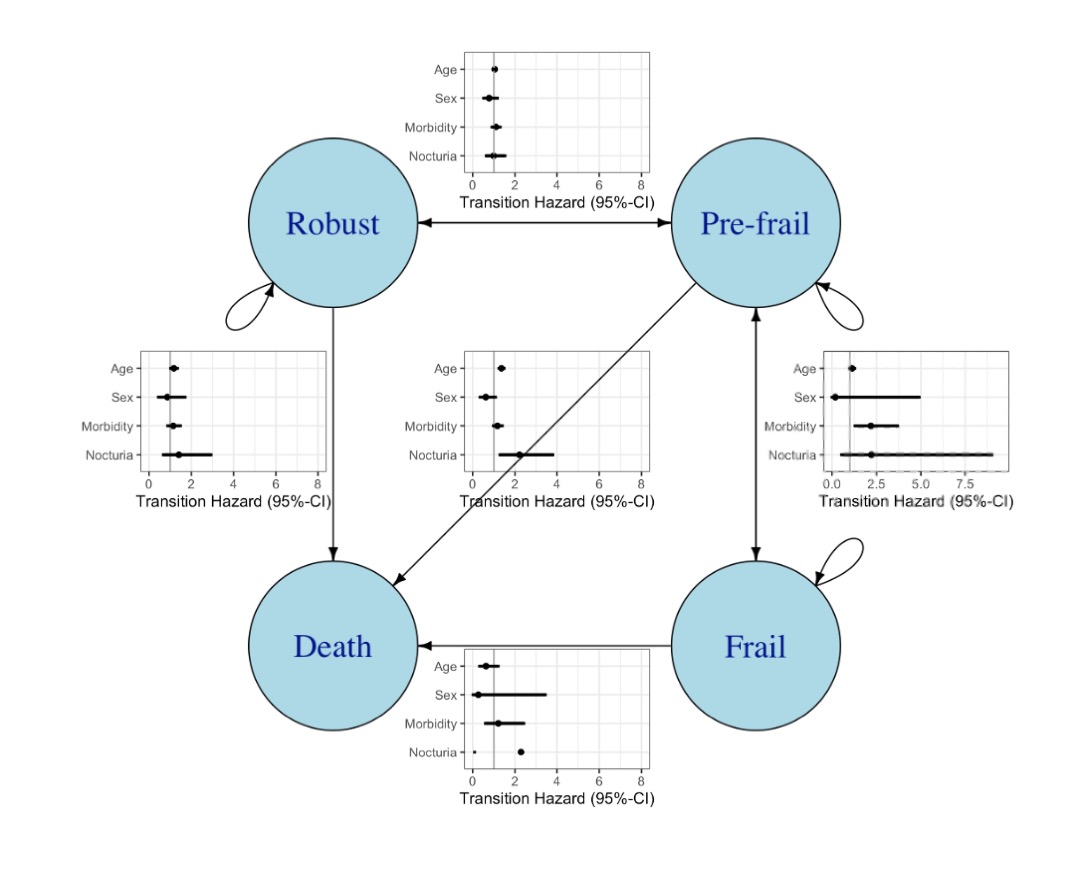


**Supplementary Figure 3.** Transition graph as modelled in the multi-state model, together with estimated transition hazard ratios for the covariates for all 'downward' transitions, i.e. robust to pre-frail (top), robust to death (left), pre-frail to frail (right), pre-frail to death (middle) and frail to death (bottom).
